# Gender and Sexuality Awareness in Medical Education and Practice: Mixed Methods Study

**DOI:** 10.2196/59009

**Published:** 2024-10-08

**Authors:** Rola Khamisy-Farah, Eden Biras, Rabie Shehadeh, Ruba Tuma, Hisham Atwan, Anna Siri, Manlio Converti, Francesco Chirico, Łukasz Szarpak, Carlo Biz, Raymond Farah, Nicola Bragazzi

**Affiliations:** 1 Azrieli Faculty of Medicine Bar-Ilan University Safed Israel; 2 Department of Obstetrics and Gynecology Galilee Medical Center Nahariya Israel; 3 Department of Internal Medicine, Kaplan Medical Centre Hebrew University Rehovot Israel; 4 United Nations Educational, Scientific and Cultural Organization Chair Anthropology of Health, Biosphere and healing systems University of Genoa Genoa Italy; 5 Department of Wellbeing, Nutrition and Sport Pegaso University Naples Italy; 6 ASL Napoli 2 Nord Naples Italy; 7 Post-Graduate School of Occupational Health Università Cattolica del Sacro Cuore Rome Italy; 8 Henry JN Taub Department of Emergency Medicine, Baylor College of Medicine Houston, TX United States; 9 Institute of Outcomes Research Maria Sklodowska-Curie Medical Academy Warsaw Poland; 10 Orthopedics and Orthopedic Oncology Department of Surgery, Oncology and Gastroenterology (DiSCOG) University of Padua Padua Italy; 11 Laboratory for Industrial and Applied Mathematics Department of Mathematics and Statistics York University Toronto, ON Canada; 12 Department of Food & Drug University of Parma Parma Italy

**Keywords:** gender medicine, medical education, clinical practice, gender-sensitive care, gender awareness, sexuality awareness, awareness, medical education and practice, healthcare, patient outcomes, patient, patients, medical professionals, training, educational interventions, status-based, survey, effectiveness, medical workforce

## Abstract

**Background:**

The integration of gender and sexuality awareness in health care is increasingly recognized as vital for patient outcomes. Despite this, there is a notable lack of comprehensive data on the current state of physicians’ training and perceptions in these areas, leading to a gap in targeted educational interventions and optimal health care delivery.

**Objective:**

The study’s aim was to explore the experiences and perceptions of attending and resident physicians regarding the inclusion of gender and sexuality content in medical school curricula and professional practice in Israel.

**Methods:**

This cross-sectional survey targeted a diverse group of physicians across various specializations and experience levels. Distributed through Israeli Medical Associations and professional networks, it included sections on experiences with gender and sexuality content, perceptions of knowledge, the impact of medical school curricula on professional capabilities, and views on integrating gender medicine in medical education. Descriptive and correlational analyses, along with gender-based and medical status-based comparisons, were used, complemented, and enhanced by qualitative analysis of participants’ replies.

**Results:**

The survey, encompassing 189 respondents, revealed low-to-moderate exposure to gender and sexuality content in medical school curricula, with a similar perception of preparedness. A need for more comprehensive training was widely recognized. The majority valued training in these areas for enhancing professional capabilities, identifying 10 essential gender-related knowledge areas. The preference for integrating gender medicine throughout medical education was significant. Gender-based analysis indicated variations in exposure and perceptions.

**Conclusions:**

The study highlights a crucial need for the inclusion of gender and sexuality awareness in medical education and practice. It suggests the necessity for curriculum development, targeted training programs, policy advocacy, mentorship initiatives, and research to evaluate the effectiveness of these interventions. The findings serve as a foundation for future directions in medical education, aiming for a more inclusive, aware, and prepared medical workforce.

## Introduction

The contemporary health care landscape is undergoing a significant transformation, with a growing recognition of the importance of integrating gender and sexuality awareness into both medical education and clinical practice [1-5]. This shift in perspective acknowledges that gender and sexuality are not just marginal issues but, on the contrary, are central determinants of health outcomes [6], influencing patient care in complex and diverse ways, affecting various aspects, from the prevalence and presentation of diseases to treatment responses and patient interactions [1,6].

Despite this growing awareness, there remains a significant gap in our understanding of how well attending and resident physicians are trained in these areas [7-10]. This includes a lack of comprehensive data on the depth and breadth of their knowledge, the extent of their exposure to gender and sexuality issues during their training, and their perceptions and attitudes toward these crucial aspects of patient care [1,9]. This paucity of information is problematic because it hinders the ability of medical education institutions and health care organizations to develop targeted educational interventions [9].

Without a clear understanding and an updated picture of the current state of medical education, training, and knowledge, it becomes challenging to craft effective strategies to enhance the competencies and skills of health care providers in dealing with gender- and sexuality-related health issues [10].

Our study aims to fill this critical gap by exploring the experiences and perceptions of attending and resident physicians regarding the inclusion of gender and sexuality content in their education and subsequent professional practice. We intend to paint a clearer snapshot of the current state of awareness and understanding in the medical community. Our objective is to identify not only the strengths but also the potential areas for improvement in medical education regarding gender and sexuality. This will enable us to contribute valuable insights to the ongoing discourse on personalized and gender-sensitive health care. In doing so, we seek to influence the future direction of medical education and practice, steering it toward a more inclusive, aware, and responsive model that takes into account the diverse needs of patients. This, in turn, is expected to lead to more effective, personalized patient care, better health outcomes, and a health care system that is more attuned to the complexities of human diversity.

## Methods

### Survey Design and Participants

This study used a mixed methods, cross-sectional survey design. We targeted a diverse group of Israeli attending and resident physicians, encompassing various specializations, professional statuses, and levels of experience. The survey was distributed through multiple Israeli Medical Associations and professional networks to ensure a wide reach.

### Demographics

The demographic section of the survey covered age, sex and gender, medical specialization, professional status (attending or resident physician), and years of experience in the medical field. This information provided a sociodemographic context for the subsequent analysis.

### Survey Content

The survey was devised based on a previous systematic review of the literature [1] and consisted of several sections, each focusing on different aspects of gender and sexuality in medical education and practice.

#### Experiences With Gender and Sexuality Content

This section assessed the respondents’ exposure to and preparedness in gender and sexuality topics during their attendance in medical schools and residency training. More specifically, participants were asked whether medical school curricula and residency programs included content related to gender and sexuality. They were then asked to rate their levels of exposure to gender and sexuality content during their academic studies and residency on a Likert scale from 1 (strongly disagree) to 5 (strongly agree).

#### Perceptions of Knowledge and Tools

Respondents rated their current levels of knowledge and the adequacy of tools available to address gender and sexuality issues in their professional practice on a Likert scale from 1 (strongly disagree) to 5 (strongly agree).

#### Impact of Medical School Curricula and Residency Programs on Professional Capabilities

This part evaluated the perceived impact of gender and sexuality training on enhancing professional capabilities and identified essential areas of knowledge in this domain. Participants were asked to what extent they felt they lacked training in the field of gender and sexuality, and to what extent they believed training in these areas would contribute to their professional capabilities, using a Likert scale from 1 (no contribution) to 5 (very great extent).

#### Integration of Gender Medicine in Medical Education

Respondents shared their views on when and how gender medicine should be incorporated into medical education, with options including preclinical years, clinical years, both preclinical and clinical years, or not at all.

#### Integration of Gender Medicine in Clinical Practice

In this section, participants were asked whether they considered the patient’s sex and gender when choosing drug treatments and whether they considered the effects of treatment on the patient’s life course in relation to sex and gender, both rated on a Likert scale from 1 (strongly disagree) to 5 (strongly agree). They were also asked if they had observed differences in the presentation and nature of symptoms based on the patient’s sex and gender and whether they believed there is a distinction in treating the LGBTQI (lesbian, gay, bisexual, transgender, queer/questioning, and intersex) population in terms of common conditions and emotional impacts.

Finally, the survey inquired if participants had mentored students in the past year, with a simple yes or no response.

This comprehensive survey aimed to gather quantitative detailed information on physicians’ experiences and perceptions regarding gender and sexuality content in their education and professional practice, highlighting areas for potential improvement in medical training.

### Statistical Analysis

Descriptive statistics were used to summarize the sociodemographic data. We used correlational analysis to explore relationships between different aspects of gender and sexuality awareness and training. A gender-based analysis was conducted to discern any differences in responses based on sex and gender. In addition, we compared responses between attending physicians and residents to identify variations based on professional status. Multivariate analyses were performed to uncover associations between survey responses and demographic variables such as age, sex and gender, years of experience, and medical specialization. Effect sizes were computed as Cohen *d* and odds ratios. For all analyses, a significance level of .05 was used as the statistical threshold. To control for the increased risk of type I error due to multiple comparisons, the Bonferroni correction was applied where necessary. All statistical analyses were conducted in the open-source R environment (R Foundation for Statistical Computing).

### Qualitative Analysis

In addition to the quantitative survey, qualitative data were collected through the inclusion of 2 open-ended items within the survey itself. These items aimed to gain deeper insights into the experiences and perceptions of attending and resident physicians regarding essential gender knowledge for medical education. More specifically, the 2 open-ended items included in the survey asked the participants to select terms or concepts related to gender knowledge that they believed are essential for medical students to study.

Thematic analysis was used to analyze the qualitative data. The process involved steps, such as familiarization, coding, theme development, defining themes, and reporting. First, responses were read multiple times to become familiar with the data. Initial codes were generated by systematically identifying key terms and concepts mentioned by respondents. Codes were then grouped into potential themes based on commonalities and patterns in the responses. Each theme was defined and named, providing a detailed analysis of its significance in the context of gender and sexuality education. The final themes were integrated into a coherent narrative, illustrating the respondents’ views on essential gender knowledge for medical students.

### Ethical Considerations

The study was conducted in compliance with the ethical standards for research involving human participants. They were informed about the purpose of the study, and participation was voluntary. Informed, written consent was collected before the commencement of the study. Anonymity and confidentiality of responses were maintained throughout the study.

## Results

### Sociodemographic Data

The survey data encompassed 189 respondents with an average age of 39.8 (SD 12.1, range 22-70, median 36; Table 1). Gender distribution showed a majority of women, totaling 57.1% (108/189) of participants. In terms of medical specialization, the respondents represented 27 different fields, with internal medicine being the most common, reported by 37% (70/189) of participants. Regarding professional status, the sample was almost evenly split between attending physicians and residents, with the former category being slightly more common at 104 out of 189 (55%) instances. Concerning their tenure in the medical field across 5 distinct experience categories (0-5 years, 6-10 years, 11-15 years, 16-20 years, and more than 20 years), the survey reflected a less experienced demographic, with the 0-5 years category reported most frequently by 86 out of 189 (45.5%) respondents, thus highlighting a considerable representation of early-career physicians within the surveyed population.

**Table 1:** Demographics of the survey’s sample.

Demographic variable	Details
Sample size	189 respondents
Age	39.8 (range 22-70, median 36) years
Gender distribution	Women: 108 (57.1%) respondents
Medical specialization	27 different medical fields; most common: Internal medicine (37%)
Professional status	Attending physicians: 104 (55%) respondents; residents: 85 (45%) respondents
Years of experience	86 (45.5%) respondents with 0-5 years of experience
Mentorship	Active involvement in student mentorship reported by 65.1% of respondents

### Respondents’ Experiences With Gender and Sexuality Content in Their Education and Professional Training

The average level of exposure to gender and sexuality content during academic studies was rated at 2.03 (SD 0.98), suggesting a low-to-moderate exposure among participants. Respondents rated their academic program’s preparedness in imparting gender and sexuality awareness at an average of 1.99 (SD 1.06), indicating a similar low-to-moderate perception of preparedness. The readiness provided by specialization or residency programs had a slightly higher average rating of 2.18 (SD 1.10). Regarding current knowledge and tools to address gender and sexuality issues, the average rating was 2.74 (SD 0.96). Finally, the extent of perceived lack of training in gender and sexuality fields averaged at 3.26 (SD 1.16), suggesting that respondents generally felt a need for more training in these areas.

On average, respondents rated 2.84 (SD 1.20) on the importance of considering the patient’s sex and gender when choosing drug treatment, indicating moderate agreement and some variability in responses. When it comes to accounting for the effects of treatment on a patient’s life course in relation to sex and gender, the mean rating was higher at 3.39 (SD 1.17), suggesting a generally higher agreement on this consideration. Observations of differences in symptom presentation based on the patient’s sex and gender had a mean rating of 2.97 (SD 1.12), reflecting that the respondents somewhat agreed that they noticed such differences.

### Respondents’ Assessment of the Impact of Training in Gender and Sexuality on Professional Capabilities

The majority of respondents valued such training highly. Approximately 47.1% (89/189) believed it can contribute to a great extent, while 20.1% (38/189) felt it can contribute to a very great extent. A further 16.4% (31/189) saw it as moderately impactful, whereas only 9% (17/189) considered its potential contribution small. Notably, 6.3% (12/189) perceived no contribution from this training. A small fraction of respondents (2/189, 1.1%) had mixed views. Overall, these findings indicate a strong consensus on the positive impact of gender and sexuality training in enhancing professional capabilities (Table 2).

**Table 2:** Major findings of the survey.

Major finding	Details
Exposure to gender and sexuality content	Low-to-moderate exposure during academic studies (Average rating: 2.03 out of a Likert scale from 1 to 5)
Preparedness to gender-sensitive care	Low-to-moderate perception of preparedness (Academic: 1.99; Specialization/Residency: 2.18, out of a Likert scale from 1 to 5)
Perceived need for training	General consensus on the need for more training (Average lack of training rating: 3.26, out of a Likert scale from 1 to 5)
Impact on professional capabilities	Majority see training as beneficial (47.1%=great extent; 20.1%=very great extent; 16.4%=moderate extent)
Essential gender-related knowledge areas	Ten areas identified, including patriarchy, LGBTQI^a^ awareness, gender awareness, sexual and domestic violence, gender-specific diseases and symptoms, pharmacology and gender differences, treatment compliance and gender, psychological and social effects of gender, and sex and gender-aware research; Table 3 for further details
Preference for integration of gender medicine	Majority prefer integration throughout medical education (Preclinical and clinical years: 55.6%)

^a^LGBTQI: lesbian, gay, bisexual, transgender, queer/questioning, and intersex.

According to the respondents, this training should cover 10 essential gender-related knowledge areas, as reported in Table 3.

**Table 3:** Ten essential gender-related knowledge areas that should be covered by the training, according to survey’s participants.

Gender-related knowledge area	Brief description
Patriarchy	Understanding the social organization where power is primarily held by men and its impact on health care access and treatment outcomes, which is crucial to recognize how patriarchal structures can affect both patient care and the work environment in health care settings
LGBTQI^a^ awareness	Knowledge about the health needs and challenges faced by the LGBTQI community, which includes understanding diverse sexual orientations and gender identities, and how these factors influence health risks, disease prevalence, and access to health care
Gender awareness	Recognizing and addressing gender biases and stereotypes in health care, which involves understanding how societal gender roles and expectations can impact health and health care delivery
Sexual violence	Awareness of the medical, psychological, and social implications of sexual violence, which includes understanding how to provide sensitive and appropriate care to survivors
Domestic violence	Recognizing signs of domestic violence and understanding its health implications, which also involves knowing how to provide support and resources to survivors
Gender-specific diseases and symptoms	Understanding differences in disease presentation, symptom onset, and diagnosis between sexes and genders, which is essential for accurate diagnosis and effective treatment
Pharmacology and gender differences	Acknowledging how drugs may affect sexes and genders differently in terms of efficacy, side effects, and treatment response, which is vital for personalized medicine
Treatment compliance and gender	Recognizing that gender can influence treatment adherence and response, with factors such as societal roles, communication styles, and access to health care varying between genders and impacting treatment outcomes
Psychological and social effects of gender	Understanding the broader psychological and social implications of gender on health, which includes the impact of gender roles, expectations, and discrimination on mental health and social well-being
Sex and gender-aware research	Promoting and using research that takes into account sex and gender differences, ensuring that medical knowledge and practice are based on inclusive and comprehensive data

^a^LGBTQI: lesbian, gay, bisexual, transgender, queer/questioning, and intersex.

According to the respondents, these topics provide a broad and nuanced understanding of how sex and gender affect health and health care, equipping medical students to deliver more compassionate, informed, and effective care to all patients, regardless of their sex and gender.

Finally, respondents had various opinions on when gender medicine should be incorporated into medical education. The majority believed it should be taught during both preclinical and clinical study years, with 55.6% (105/189) respondents endorsing this approach. Furthermore, 50 respondents, out of 189, felt it should be specifically included in the clinical study years (26.5%), and 23 participants argued for its introduction in the preclinical years (12.2%). A minority of 3.7% (7/189) subjects believed there was little need to teach gender medicine. There are also a few isolated responses that combine these categories or indicate no need at all for such education, each with 1 respondent. Overall, this distribution indicates a strong preference for integrating gender medicine throughout the entire span of medical education, with a significant emphasis on its presence in both foundational and advanced stages of the medical curriculum.

Among the 189 survey participants, 123 respondents indicated that they have been mentoring students in the past year (65.1%), while 66 respondents have not engaged in student mentorship during that time (34.9%). This suggests a significant portion of the respondents are actively involved in the mentorship and educational development of students.

### Correlations and Trends: Insights From Correlational Analysis

There was a strong correlation (*r*=0.70) between respondents’ perceptions of their academic program’s preparation in terms of gender and sexuality awareness and their views on the preparation provided by their specialization and residency program. Furthermore, respondents’ levels of exposure to gender and sexuality content in their academic program strongly correlated (*r*=0.68) with their perception of how well the program prepared them in these areas. There was a moderate correlation (*r*=0.48) between how well respondents feel their specialization and residency program prepared them and their current perception of having sufficient knowledge and tools to deal with gender and sexuality issues in their field. Furthermore, respondents who felt their academic program prepared them well in gender and sexuality awareness also tend to feel they currently have sufficient knowledge and tools in this area, with a moderate correlation (*r*=0.40).

### Gender-Based Analysis

The analysis based on gender reveals null-to-small effect sizes, with only 1 medium effect size concerning the sex- and gender-specific choice of a treatment. Men reported a higher level of exposure to gender and sexuality content during their academic studies (mean 2.23, SD 1.06) compared with women (mean 1.87, SD 0.89; *d*=0.38). Similarly, they also rated their academic study program’s preparation in gender and sexuality awareness higher (mean 2.22, SD 1.15 for men vs mean 1.82, SD 0.97 for women; *d*=0.38). In terms of how well respondents felt their specialization and residency program prepared them in gender and sexuality awareness, men’s responses were only slightly higher (mean 2.28, SD 1.15) than women’s (mean 2.10, SD 1.05; *d*=0.17; not statistically significant). When asked if they currently have sufficient knowledge and tools to deal with issues of gender and sexuality in their field, men’s responses were on average comparable (mean 2.79, SD 1.02) with those of women (mean 2.70, SD 0.92; *d*=0.09; not statistically significant). Regarding the extent to which they feel they lack training in the field of gender and sexuality, men had a lower average (mean 3.10, SD 1.21) than women (mean 3.38, SD 1.12; *d*=–0.24), suggesting that women perceive a greater need for training in these areas. Regarding the consideration of patient’s sex and gender in drug treatment, men reported an average score of 3.10 (SD 1.22), higher than those reported by women (mean 2.64, SD 1.16), with *d*=0.39. When choosing the treatment, men were more likely to take into account its effects on the patient’s life course in relation to sex and gender (mean 3.85, SD 1.02 vs mean 3.07, SD 1.16; *d*=0.71). When questioned about the observation of differences in the presentation and nature of symptoms based on patient’s sex and gender, the responses from both men and women participants were similar (mean 2.99, SD 1.18 vs mean 2.96, SD 1.08; *d*=0.02). Similarly, recognition of the unique health care needs and challenges faced by LGBTQI individuals did not differ between men and women (mean 3.11, SD 1.31 vs mean 3.21, SD 1.22; *d*=–0.08).

In terms of subjects mentioned by respondents, men were more likely to mention certain topics like “domestic violence,” “homophobia,” “LGBTQI awareness,” and “gender awareness.” Other subjects such as differences in symptom onset and diagnosis of gender-specific diseases, topics related to pharmacology, treatment compliance, gender aspects of heart health, disease prevention, psychological and social effects, and feminism were more common among men respondents. Among women, respondents’ topics covered more medically gender-related subjects, such as sexually transmitted infections, sexual education, and safer sex, among others.

To get more insights about gender-specific differences in the responses, further gender-based analyses were conducted. No gender-specific differences could be found in terms of age (mean 41.0, SD 14.3 vs mean 38.9, SD 10.1; *P*=.23) and the status of the respondent, that is, attending versus resident (χ^2^_1_=0.02, *P*=.90). On the contrary, there were some gender imbalances concerning the different medical specializations of the respondents (χ^2^_26_=36.76, *P*=.08). In particular, in the field of pediatrics all subjects were practically women (10 vs 1; *P*=.03 at the post hoc test), and in the field of internal medicine, men were overrepresented compared with women (42 vs 28; *P*<.001 at the post hoc test). In terms of years of practice and experience, some slight gender imbalances could be noted (χ^2^_4_=6.94, *P*=.14), with men being overrepresented in the category “more than 20 years” (24 vs 16; *P*=.02 at the post hoc test). Finally, women were more likely to report not having mentored students in the last year (χ^2^_1_=3.76, *P*=.05, with an odds ratio of 1.84 [95% CI 0.99-3.43]).

### Medical Status-Based Analysis

The comparison between attending physicians and residents yields the following insights. On average, attending physicians reported a lower level of exposure to gender and sexuality content during academic studies (mean 1.88) compared with residents (mean 2.21). Similarly, they rated their academic study program’s preparation in gender and sexuality awareness lower (mean 1.86 for attending doctors vs 2.16 for residents). Attending physicians also rated the preparation provided by their specialization and residency program slightly higher (mean 2.21) than residents did (mean 2.14). In assessing whether they have sufficient knowledge and tools to deal with issues of gender and sexuality, attending physicians’ average response was higher (mean 2.87) compared with residents (mean 2.59). When it comes to the extent of lacking training in gender and sexuality, attending physicians feel slightly less deficient (mean 3.20) than residents (mean 3.33), indicating that residents may perceive a greater need for training in these areas.

The comparison among the different medical specializations revealed that respondents in the field of internal medicine perceived themselves as relatively well-prepared or exposed to gender and sexuality topics.

### Multivariate Analysis

At the multivariate analysis, the reported level of exposure to gender and sexuality content during academic studies was associated with gender (*F*_2,186_=8.89, *P*=.003), with women reporting lower exposure than men (β=–.46, 95% CI –0.77 to –0.16). Similarly, perceived academic preparedness in terms of gender and sexuality awareness was found to be associated with gender (*F*_2,186_=7.33, *P*=.007), with women scoring lower than men (β=–.43, 95% CI –0.74 to –0.12), while thinking of currently having sufficient knowledge and tools to deal with issues of gender and sexuality in one’s field was associated with years of experience in a statistically significant way (*F*_5,183_=2.48, *P*=.045 at the ANOVA omnibus test). In particular, the category “over 20 years” versus “0-5 years” was more likely to report a higher score (β=1.30, 95% CI 0.27-2.33; *P*=.014). The perceived lack of training in the field of gender and sexuality was found to be associated with medical status (*F*_2,186_=4.06, *P*=.045 at the ANOVA omnibus test), with residents scoring higher than attending doctors (β=.54, 95% CI 0.01-1.08). The perceived impact of training in gender and sexuality on professional skills was once again associated with gender (*F*_2,186_=4.89, *P*=.028), with women reporting greater perceived impact (β=.34, 95% CI 0.04-0.65) than men. Accounting for the person’s sex and gender in the choice of the treatment was associated with the gender (*F*_2,186_=5.26, *P*=.023), with women reporting this practice less (β=–.37, 95% CI –0.68 to –0.05) than men. When choosing the treatment, taking into account its effects on the patient’s life course in relation to sex and gender was associated with gender (*F*_2,186_=17.12, *P*<.001), medical specialization (*F*_27,161_=19.62, *P*<.001), and increasing years of practice and experience (*F*_5,183_=2.21, *P*=.07). This practice was less reported by women (β=–.54, 95% CI –0.79 to –0.28), and doctors non specialist in internal medicine (β=–.74, 95% CI –1.07 to –0.41). No significant predictors could be found for the other items of the questionnaire.

### Qualitative Analysis

Participants highlighted 10 essential gender-related knowledge areas that should be covered by training, as identified by survey participants. First, understanding patriarchy is crucial for recognizing how power dynamics, predominantly controlled by men, can impact health care access and treatment outcomes. This knowledge helps in identifying the influence of patriarchal structures on both patient care and the work environment in health care settings. Awareness of LGBTQI health needs is also essential, encompassing knowledge about diverse sexual orientations and gender identities, and their influence on health risks, disease prevalence, and access to health care. Recognizing and addressing gender biases and stereotypes in health care, known as gender awareness, involves understanding how societal gender roles and expectations affect health and health care delivery. Awareness of sexual violence includes understanding its medical, psychological, and social implications to provide sensitive and appropriate care to survivors. Similarly, recognizing signs of domestic violence and understanding its health implications is vital, along with knowing how to provide support and resources to survivors. In addition, understanding gender-specific diseases and symptoms is essential for accurate diagnosis and effective treatment, as is acknowledging how drugs may affect sexes and genders differently in terms of efficacy, side effects, and treatment response. Recognizing that gender can influence treatment adherence and response is important, with factors such as societal roles, communication styles, and access to health care varying between genders. Understanding the broader psychological and social effects of gender on health includes considering the impact of gender roles, expectations, and discrimination on mental health and social well-being. Finally, promoting and using sex- and gender-aware research ensures that medical knowledge and practice are based on inclusive and comprehensive data, leading to improved health care outcomes for all sexes and genders.

## Discussion

### Principal Findings

This survey offered a rich and nuanced view of physicians’ experiences and perceptions related to gender and sexuality in their education and practice. The demographic data revealed an average respondent age of nearly 40 years, with a notable majority of women. Diversity was evident in their medical specializations, with internal medicine emerging as the most common field, whereas the professional status of the respondents was well-balanced between attending physicians and residents, although slightly skewed toward the former. A significant portion of the survey population was relatively new to the medical field, emphasizing the presence of early-career physicians.

In terms of their experiences with gender and sexuality content, the data suggested that the exposure during academic studies was generally low to moderate. This was mirrored in their perception of preparedness in these areas, indicating a gap in the curriculum.

The sociological landscape of LGBTQI rights in Israel has been marked by both significant progress and notable contradictions. Since the early 2000s, there have been considerable advancements for LGBTQI individuals. However, progress has been uneven, especially for the transgender community, which continues to face significant discrimination, violence, and material disadvantages. In key institutions like health care and education, LGBTQI individuals encounter barriers that reflect broader societal tensions. Privatization and economic disparities exacerbate these challenges, particularly for those without the resources to navigate these systems effectively [11].

Our survey findings can be interpreted and discussed against this framework. However, they are challenging to directly compare due to the scarcity of research in Israeli contexts, which is predominantly limited to specific populations, such as physiotherapy students [12]. On the other hand, the findings well align with the broader trends identified in the literature, emphasizing a widespread issue in medical education regarding the adequacy of training on gender and sexuality. According to a survey by Obedin-Maliver et al [13], medical schools in the United States and Canada devote a small amount of time in their curricula to LGBTQI health and other topics related to sexuality, indicating a need for more comprehensive education in these areas. This survey was conducted more than a decade ago (between May 2009 and March 2010) and replicated recently, finding that, while the median time allocated to LGBTQI health-related topics increased in US and Canadian undergraduate medical education institutions, the scope, effectiveness, and quality of this instruction varied significantly. Despite the rise in hours, the total remains below the number recommended by the Association of American Medical Colleges (AAMC) for LGBTQI health competencies [14].

Of note, when it comes to the application of this knowledge in professional practice, there was a moderate level of self-assessed competence, coupled with a general consensus on the need for more comprehensive training. The importance of considering the patient’s sex and gender in treatment decisions showed moderate agreement among the respondents, with a slightly higher emphasis on the impact of treatment in the context of the patient’s sex and gender. The impact of training in gender and sexuality on professional capabilities was largely acknowledged by the majority. This aligns with studies suggesting that insufficient training on gender and sexuality issues can lead to lower confidence among physicians when addressing the health needs of LGBTQI patients and other gender-diverse populations. For instance, a study by Marr et al [15] highlighted that many medical residents feel unprepared to provide high-quality care to LGBTQI patients, mirroring our survey’s findings on the perceived preparedness gap.

Of note, our survey’s participants expressed confidence in their ability to handle gender and sexuality issues in clinical practice, despite a low self-reported exposure to gender and sexuality content during their medical training. This apparent paradox, where a curricular gap was identified but respondents still felt prepared to deliver care, has been observed in other studies. What is particularly intriguing in our study is the differentiation between residents and fully licensed physicians, with responses further stratified by years of practice. These data seem to suggest a pattern; the longer a physician has been in practice, the less gender and sexuality content they recall from their training, yet the more confident they feel in their knowledge and ability to address these issues. This pattern could point to several possibilities. It may suggest that while formal education on gender and sexuality issues is lacking, the day-to-day experiences and challenges of medical practice provide ample opportunities for physicians to develop the necessary competencies. Alternatively, it could imply that confidence increases with experience, even if knowledge gaps persist, potentially leading to overconfidence in areas where additional training would be beneficial. This distinction between learning through experience *versus* feeling prepared due to increased confidence is a critical area for further exploration, as it has significant implications for medical education and ongoing professional development.

Furthermore, the respondents pinpointed 10 critical areas of gender-related knowledge, encompassing a broad spectrum from LGBTQI awareness to the specifics of gendered pharmacology, pointing to the multifaceted nature of sex and gender in medical practice. In discussing the incorporation of gender medicine into medical education, there was a clear preference for its integration across all stages of learning, reflecting a progressive approach toward medical training. The data also highlighted active involvement in student mentorship by a substantial number of respondents. The literature increasingly supports the integration of gender medicine and education on sexuality and gender diversity throughout the entire medical education continuum, from undergraduate education to continuing medical education for practicing physicians. This approach is advocated to ensure that medical doctors are well-equipped to meet the diverse needs of all patients, recognizing the significant role of sex and gender in health outcomes. For example, a consensus statement by the AAMC on the inclusion of gender awareness and LGBTQI health in medical education curriculum frameworks emphasizes the need for longitudinal integration rather than isolated modules or electives [16,17].

Furthermore, the survey revealed intriguing correlations, particularly between perceptions of academic program preparation and specialization and residency program views on gender and sexuality awareness. The gender-based analysis presented a complex picture, with variations in exposure and perceptions between men and women. Certain topics showed gender imbalances, while others exhibited more parity. Comparing attending physicians and residents, differences emerged in their perceptions of exposure to and preparedness in gender and sexuality content, suggesting variations in training across different stages of medical careers. The multivariate analysis further unraveled associations between various factors such as gender, years of experience, and medical status in relation to the survey responses. Similarly, a survey [18] conducted in Taiwan identified several shortcomings in present medical education and the lack of readiness among medical students and trainees to offer improved care for LGBTQI individuals.

In summary, this survey underscored the growing recognition of gender and sexuality as pivotal components in medical education and practice. It highlighted existing gaps in training and varying perceptions based on demographic and professional factors, pointing toward a need for a more inclusive and comprehensive approach in medical training and practice.

This survey offers valuable guidance for medical teachers and institutional stakeholders on developing and applying effective curricula and training programs, as well as faculty development initiatives. These strategies should aim to furnish medical students and trainees with the self-awareness and skills necessary to deliver gender-sensitive care, including comprehensive care to sexual and gender minorities, align with societal advancements, and advance health equity for a broader range of patients.

Effective communication is crucial for medical doctors, involving active listening, clear explanations, recognition of nonverbal cues, and patient education. By actively listening, health care providers can fully understand patients’ symptoms and concerns, leading to more accurate diagnoses and tailored treatments. Clear explanations about diagnoses and treatment options ensure patients can make informed decisions. Recognizing and responding to nonverbal cues enhance understanding and trust, while effective patient education ensures patients comprehend their health conditions and necessary treatments. Cultural competence is essential, requiring awareness of diverse cultural backgrounds, including values and beliefs. Sensitivity to cultural differences and avoiding stereotypes help build trust and provide respectful care. Adapting health care practices to meet cultural needs improves health outcomes and patient satisfaction. Ongoing cultural competence training enhances inclusive care. Empathy involves understanding and valuing patients’ feelings and experiences and building therapeutic relationships. Providing compassionate care alleviates anxiety and improves the health care experience. Offering emotional support and reassurance is crucial, and reflective practice helps physicians improve empathetic interactions. Navigating complex social and ethical considerations related to gender and sexuality is vital in gender-based medicine. Recognizing and respecting diverse gender identities ensures all patients receive appropriate care. Implementing inclusive practices, such as using correct pronouns and offering gender-neutral facilities, supports patient well-being. Addressing ethical dilemmas requires careful consideration of patient autonomy and confidentiality. Staying informed about gender issues, advocating for patients’ rights, and working to eliminate health care disparities are integral to ethical medical practice. These competencies—effective communication, cultural competence, empathy, and navigating gender and sexuality issues—are fundamental for medical doctors to provide comprehensive, sensitive, and effective care. Ensuring all patients feel understood, respected, and valued is the cornerstone of excellent gender-based medicine.

A further point that should be stressed is that our findings revealed significant variations in exposure and preparedness between men and women concerning gender and sexuality content in medical education. Men reported a higher level of perceived preparedness in dealing with gender and sexuality issues than women. This discrepancy highlights a crucial point often overlooked in discussions about educational interventions, that is, those in positions of privilege (in this case, men) may report more comfort and may not perceive the existing gaps as those in less privileged positions (women). Men’s higher self-reported comfort could stem from their generally more prominent status within the medical community, which may afford them more confidence in professional settings. Conversely, women, who historically and structurally face more barriers in the medical field, may experience and recognize these gaps more acutely. This perception gap is critical as it underscores the need for more targeted and inclusive educational programs that not only address the specific needs of female physicians but also raise awareness among male physicians about these disparities. In addition, it is essential to acknowledge the role of implicit biases and structural inequalities that contribute to these differing perceptions. Training programs must be designed to bridge this gap by fostering an environment where both male and female physicians can gain a more balanced and comprehensive understanding of gender and sexuality issues. This approach can lead to a more equitable and effective health care delivery system, where all practitioners are equally prepared to address the diverse needs of their patients. By incorporating these considerations into the development of medical curricula and professional training, we can work toward reducing the perception and comfort gap between male and female physicians, ultimately leading to improved patient outcomes and a more inclusive medical community.

### Future Directions

The World Federation for Medical Education (WFME) sets global standards for quality improvement in medical education. These standards include explicit requirements for integrating gender and sexuality education into medical school curricula. The WFME’s standards ensure that medical schools worldwide provide education that prepares physicians to address diverse patient needs, including those related to gender and sexuality. Our study’s findings indicate a significant gap in the integration of gender and sexuality content within medical education, highlighting a discrepancy between current practices and the WFME’s curricular requirements. As such, the findings of this survey highlight the need for a comprehensive overhaul of medical education curricula. Future efforts should focus on integrating gender and sexuality content more thoroughly and consistently across all stages of medical training. This includes both preclinical and clinical years, ensuring that medical doctors are equipped with the necessary knowledge and skills from the onset of their careers.

Given the reported gap in preparedness and exposure, there is a clear need for targeted training programs that address specific areas of gender and sexuality in health care. These programs should cover the 10 critical areas identified by respondents, ranging from LGBTQI awareness to gender-specific diseases and symptoms.

Further research is necessary to continuously monitor and evaluate the effectiveness of implemented educational strategies. Longitudinal studies could be beneficial in assessing the impact of improved gender and sexuality training on health care outcomes. In addition, research should explore the evolving needs and perceptions of medical residents and practicing physicians in these areas.

The study’s results can be used to advocate for policy changes at institutional and national levels. This involves lobbying for mandatory inclusion of gender and sexuality topics in medical education accreditation standards and continuous professional development requirements.

Furthermore, mentorship programs that emphasize gender and sexuality awareness should be encouraged. This point is crucial and experienced medical doctors who are well-versed in these topics should mentor younger colleagues, fostering a culture of continuous learning and sensitivity toward these issues. Efforts should be made to promote diversity and inclusion within the medical community, addressing gender imbalances in various medical specializations and ensuring that medical education and practice are inclusive of all sexes, genders, sexual orientations, and gender identities.

The latest technological advancements can be leveraged. Using, for instance, virtual reality and e-learning platforms, can provide innovative ways to teach and engage medical students and practicing doctors in gender and sexuality topics [19,20]. This approach can supplement traditional learning methods and offer flexible training opportunities. Future studies investigating the effectiveness of these technological methods in gender medicine education are crucial as they would help in understanding how well these technologies enhance learning outcomes, their impact on the practical skills of medical doctors, and how they compare with traditional teaching methods. Implementing technology in medical education, especially for topics like gender and sexuality, represents a significant step forward in creating a more informed and sensitive health care environment.

### Conclusions

This study underscores the critical need for integrating gender and sexuality awareness into medical education and practice, finding that, despite the recognized importance, there is a notable gap in the current training and preparedness of medical residents and practicing physicians in these areas. The survey results reveal a consensus on the necessity for more comprehensive training, reflecting the evolving landscape of health care where gender and sexuality play a significant role in patient care and outcomes. The variations in exposure and perceptions based on gender, professional status, and years of experience highlight the diversity of learning and training needs within the medical community. This calls for a tailored approach in educational interventions, ensuring that they are relevant and effective for various groups within the medical profession.

Overall, the study contributes significantly to the ongoing discourse on personalized, gender-sensitive health care, by providing valuable insights for educators, policy makers, and health care providers, emphasizing the need for a more inclusive, aware, and well-prepared medical workforce to cater to the diverse health care needs of the population.

## Abbreviations

AAMC: Association of American Medical Colleges
LGBTQI: lesbian, gay, bisexual, transgender, queer/questioning, and intersex
WFME: World Federation for Medical Education
